# The Aryl Hydrocarbon Receptor Suppresses Chronic Smoke-Induced Pulmonary Inflammation

**DOI:** 10.3389/ftox.2021.653569

**Published:** 2021-07-06

**Authors:** Necola Guerrina, Hussein Traboulsi, David H. Eidelman, Carolyn J. Baglole

**Affiliations:** ^1^Research Institute of the McGill University Health Centre, Montreal, QC, Canada; ^2^Department of Medicine, McGill University, Montreal, QC, Canada; ^3^Deaprtment of Pathology, McGill University, Montreal, QC, Canada; ^4^Department of Pharmacology and Therapeutics, McGill University, Montreal, QC, Canada

**Keywords:** aryl hydrocarbon receptor, cigarette smoke, inflammation, lungs, multinucleated (foreign body) giant cell

## Abstract

The aryl hydrocarbon receptor (AhR) is a ligand-activated transcription factor expressed in the lungs that is activated by numerous xenobiotic, endogenous and dietary ligands. Although historically the AhR is known for mediating the deleterious response to the environmental pollutant dioxin, emerging evidence supports a prominent role for the AhR in numerous biological process including inflammation. We have shown that the AhR suppresses pulmonary neutrophilia in response to acute cigarette smoke exposure. Whether the AhR can also prevent lung inflammation from chronic smoke exposure is not known but highly relevant, given that people smoke for decades. Using our preclinical smoke model, we report that exposure to chronic cigarette smoke for 8-weeks or 4 months significantly increased pulmonary inflammation, the response of which was greater in *Ahr*^−/−^ mice. Notably, there was an increased number of multinucleated giant cells (MNGCs) in smoke-exposed *Ahr*^−/−^ mice without a change in cytokine levels. These data support a protective role for the AhR against the deleterious effects of cigarette smoke, warranting continued investigation into its therapeutic potential for chronic lung diseases.

## Introduction

The aryl hydrocarbon receptor (AhR) is a transcription factor that is activated in response a multitude of low molecular weight compounds of both exogenous and endogenous origins; these include dioxins, polycyclic aromatic hydrocarbons (PAHs), plant polyphenols and tryptophan metabolites (Abel and Haarmann-Stemmann, 2010; Quintana, 2013). These bind to the ligand-binding domain located on the amino terminal of the cytoplasmic AhR. Upon entering the nucleus, the AhR dissociates from its chaperone proteins prior to heterodimerization with the aryl hydrocarbon receptor nuclear translocator (ARNT). This AhR-ARNT complex then binds to the xenobiotic response element in the promoter region of AhR target genes, the prototypical of which is cytochrome P450 (CYP) CYP1A1 (Nukaya et al., 2009).

Historically, the AhR is best-known for its ability to mediate the deleterious effects of the man-made toxicant 2,3,7,8-tetrachlorodibenzo-p-dioxin (TCDD; dioxin) (Bock, 1994; Fernandez-Salguero et al., 1996; Mimura et al., 1997). However, the AhR is evolutionarily conserved amongst multicellular organisms (Williams et al., 2014) and is highly expressed in the skin, gut, liver and lungs (Esser and Rannug, 2015). The lungs are continuously exposed to the external environment and are particularly sensitive to environmental toxicants, including those derived from air pollution (ambient, biomass) and cigarette smoke. Cigarette smoke is also a leading cause of preventable death and is the main risk factor for the development of lung cancer and chronic obstructive pulmonary disease (COPD). The pathogenesis of these diseases is typified by chronic inflammation, initiated and perpetuated by inhalation of cigarette smoke. Cigarette smoke is a complex mixture that contains chemicals that activate the AhR including the PAH benzo[*a*]pyrene (B[*a*]P). However, the AhR has emerged as a critical component of immunity, dampening the severity of diseases associated with chronic inflammation such as rheumatoid arthritis (Rosser et al., 2020; Nehmar et al., 2021), inflammatory bowel disease (Riemschneider et al., 2021), asthma (Chang et al., 2020), periodontitis (Huang et al., 2019), psoriasis (Di Meglio et al., 2014) and multiple sclerosis (Abdullah et al., 2019). We have previously shown that the AhR attenuates pulmonary neutrophilia in response to acute and sub-chronic cigarette smoke exposure (Thatcher et al., 2007; de Souza et al., 2014; Rico de Souza et al., 2021) and prevents the development of a COPD-like phenotype (Guerrina et al., 2021). However, it remains unknown whether the AhR can reduce inflammation caused by chronic cigarette smoke exposure, findings that are relevant given that people who develop COPD often smoke for decades. Therefore, we utilized our preclinical cigarette smoke model to study whether the AhR protects against chronic smoke-induced pulmonary inflammation. This work enhances our understanding of the AhR in protecting against the harmful effects of long-term cigarette smoking, which still claims the lives of millions of people each year.

## Methods

### Animals

*Ahr*-knockout (*Ahr*^−/−^) C57BL/6 mice were obtained from Jackson Laboratory (Strain B6.129-Ahr^tm1^/J; Bar Harbor, ME). *Ahr* heterozygous C57BL/6 mice (*Ahr*^+/−^) were bred in house and maintained on an *ad libitum* diet. *Ahr*^+/+^ or *Ahr*^+/−^ mice do not exhibit any difference in the ability to be activated by AhR ligands or cigarette smoke and are used interchangeably as AhR-expressing mice (Thatcher et al., 2007; Baglole et al., 2008; Zago et al., 2013; de Souza et al., 2014), rendering mice of the *Ahr*^+/−^ genotype as littermate controls.

### Preclinical Cigarette Smoke Model

Age and gender-matched *Ahr*^−/−^ and *Ahr*^+/−^ mice were exposed to cigarette smoke using a whole-body exposure system (InExpose; SCIREQ Inc., Montreal, Canada) for up to 4 months as previously described (Thatcher et al., 2007; Zago et al., 2013; de Souza et al., 2014). Mice were between 8 and 12 weeks at the start of the experiments, corresponding to ~21 years in humans (Dutta and Sengupta, 2016). Male mice were used for the 8-week experiments (4–5 mice per group) and both male and female mice for the 4-month experiment (9–11 mice per group). Considering the lifespan between mice and humans, this corresponds to people who smoke for ~6–12 years (Dutta and Sengupta, 2016). All animal procedures were approved by the McGill University Animal Care Committee (Protocol Number: 5933) and were carried out in accordance with the Canadian Council on Animal Care.

### Tissue Harvest and Bronchoalveolar Lavage (BAL) Collection

Following the last exposure, mice were anesthetized with Avertin (2,2,2-tribromoethanol, 250 mg/kg ip; Sigma-Aldrich, St Louis, MO) and sacrificed by exsanguination. The lungs were removed and lavaged twice with 0.5 ml of PBS. After the bronchoalveolar lavage (BAL) fluid was centrifuged, the supernatant was removed, and the BAL cell pellets were resuspended in PBS. Total cell counts were determined using a hemocytometer. Differential cell counts were performed after cytospin slide preparation (Thermo Shandon, Pittsburgh, PA) following staining with Hema-Gurr Stain (Merck, Darmstadt, Germany).

### Detection of Cytokine Levels

BAL fluid was collected as described above and stored at −80°C until used. BAL cytokine levels were evaluated using Luminex® technology (Milliplex xMAP, Millipore, Billerica, MA) for the 8-week exposure or were performed by Eve Technologies (Calgary AB).

### Statistical Analysis

Statistical analysis was performed using Prism 6-1 (La Jolla, CA). Statistical differences between group mean values were determined by 2-way analysis of variance (ANOVA) followed by the Neuman-Keuls multiple comparisons test unless otherwise indicated. In all cases, a *p* < 0.05 is considered statistically significant.

## Results

### The AhR Attenuates Chronic Cigarette Smoke-Induced Pulmonary Inflammation and Formation of Multinucleated Giant Cells

**Figure 1:**
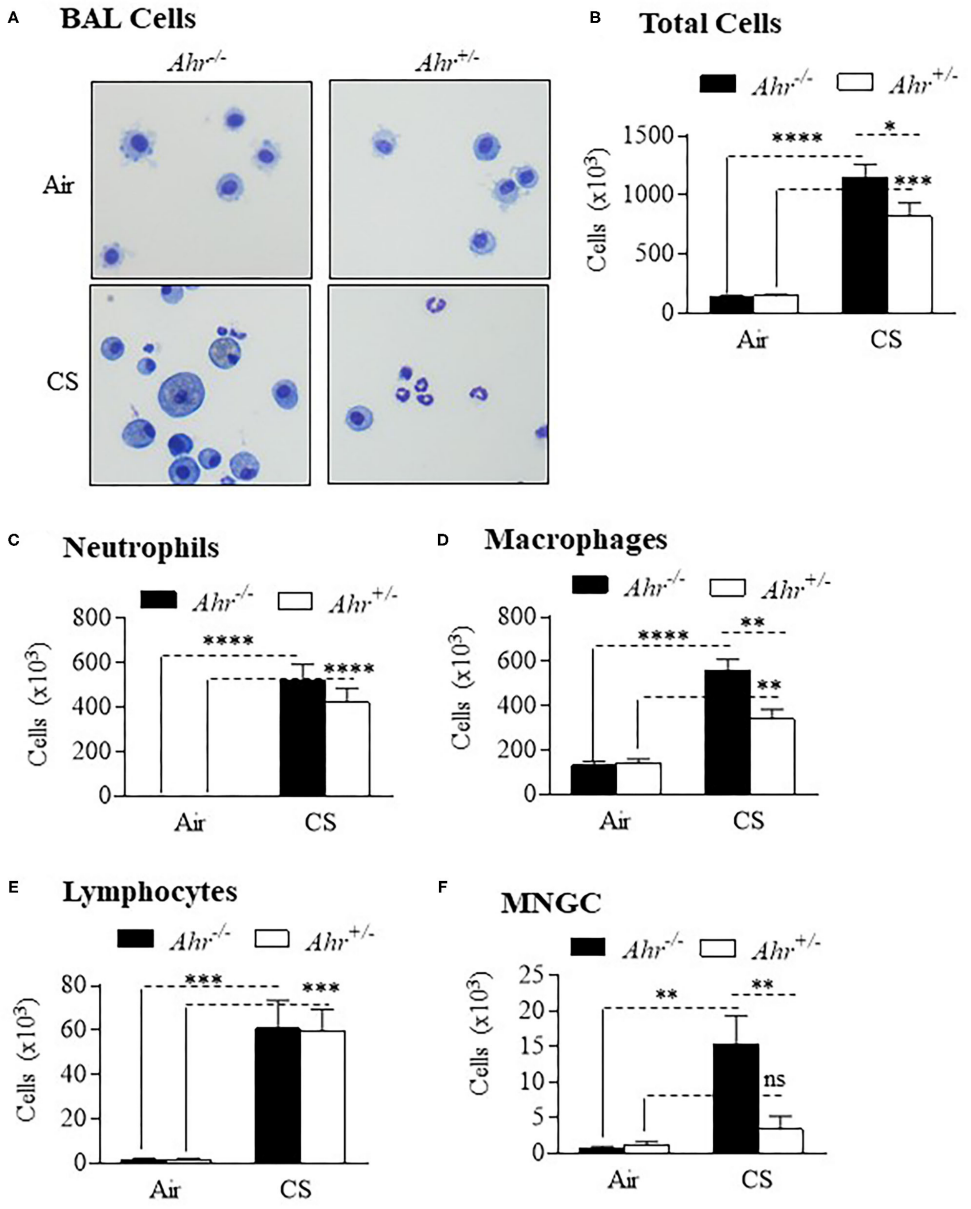
AhR deficiency results in heightened pulmonary inflammation in response to an 8-week cigarette smoke (CS) exposure regime. **(A)** BAL Cells- Representative images of cells from the BAL of *Ahr*^+/−^ and *Ahr*^−/−^ mice exposed to an 8-week CS regime. **(B)** Total Cells- There was a significant increase in cellularity in response to CS (****p < 0.0001; ***p < 0.001 compared to air-only exposure). There were significantly more BAL cells in CS-exposed *Ahr*^−/−^ mice (*p < 0.05 compared to CS-exposed *Ahr*^+/−^ mice). **(C)** Neutrophils- CS induced a significant increase in BAL neutrophils in both *Ahr*^+/−^ and *Ahr*^−/−^ mice (****p < 0.0001). **(D)** Macrophages- CS increased the number of macrophages in *Ahr*^+/−^ and *Ahr*^−/−^ mice (****p < 0.0001); macrophages were also significantly higher in CS-exposed *Ahr*^−/−^ mice (**p < 0.05); **(E)** Lymphocytes- There was a significant increase in lymphocytes in response to CS (***p < 0.001 compared to air controls. **(F)** MNGC—MNGCs were significantly increased in CS-exposed *Ahr*^−/−^ mice compared to both air-exposed *Ahr*^−/−^ mice (**p < 0.01) as well as CS-exposed *Ahr*^+/−^ mice (**p < 0.01). Results are expressed as mean ± SEM, *n* = 4–5 mice per group; 2-way ANOVA followed by the Neuman-Keuls multiple comparisons test.

To assess if the absence of the AhR was associated with an increase in inflammation from chronic smoke exposure, we first chose an 8-week cigarette smoke exposure, a time-frame that precedes the development of lung structural and functional alterations. There were significantly more cells in the BAL of smoke-exposed *Ahr*^−/−^ mice compared to *Ahr*^+/−^ mice (Figures 1A,B). Although neutrophil numbers were significantly elevated as a consequence of an 8-week cigarette smoke-exposure, there was no significant difference in BAL neutrophils between the smoke-exposed *Ahr*^−/−^ and *Ahr*^+/−^ mice (Figure 1C). While we also observed that cigarette smoke-exposed *Ahr*^−/−^ mice have significantly more macrophages (Figure 1D), there were similar levels of lymphocytes between smoke-exposed *Ahr*^−/−^ and *Ahr*^+/−^ mice (Figure 1E). However, there were significantly more multi-nucleated giant cells (MNGCs) in the BAL of *Ahr*^−/−^ mice exposed to cigarette smoke for 8 weeks (Figure 1F).

**Figure 2:**
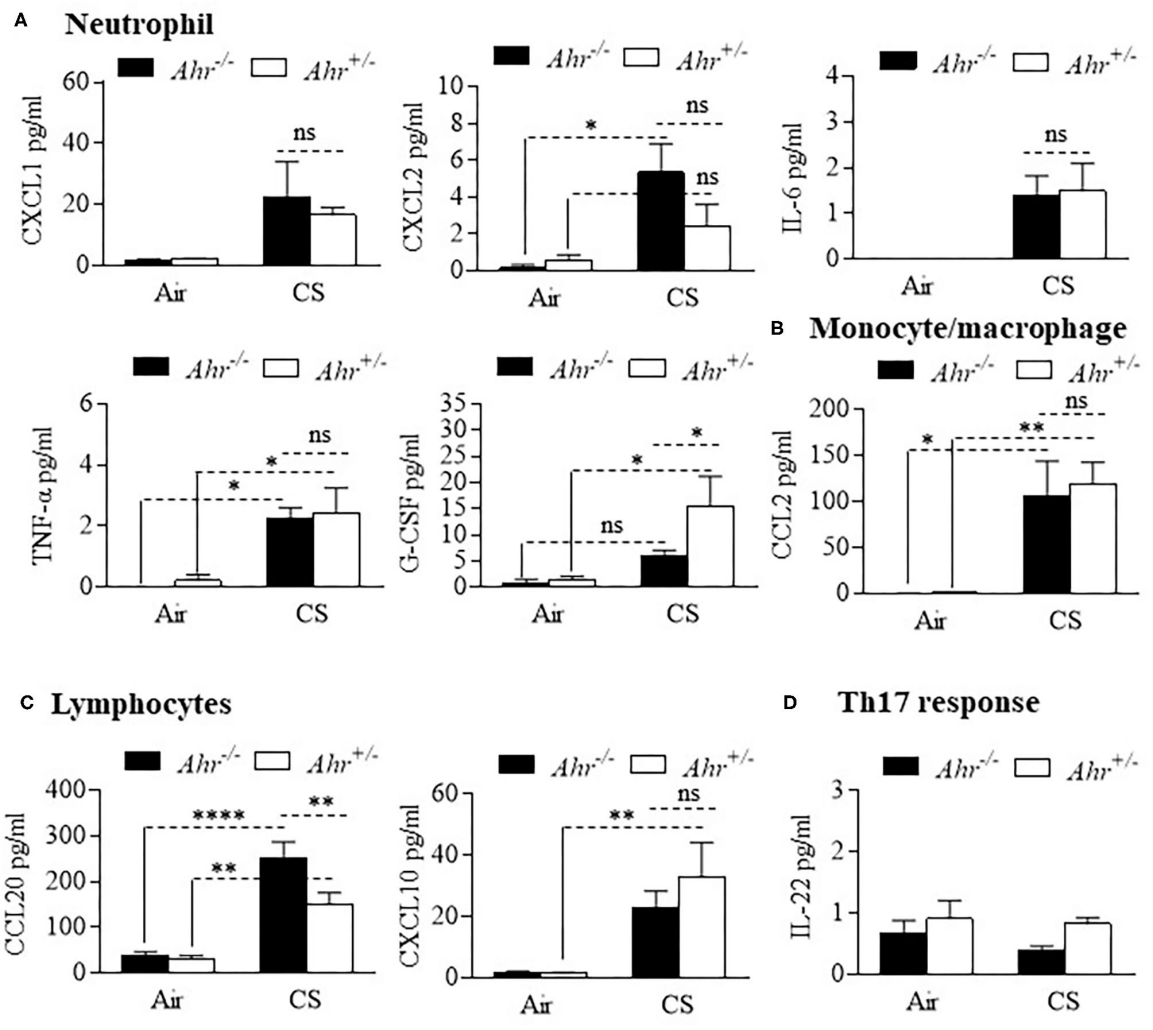
Differential analysis of BAL cytokines is similar between smoke-exposed *Ahr*^+/−^ and *Ahr*^−/−^ mice. *Ahr*^+/−^ and *Ahr*^−/−^ mice were exposed to an 8-week CS regime and cytokines analyzed in the BAL fluid by multiplex analysis. **(A)** Neutrophils- there was an overall increase in the levels of CXCL1, CXCL2, IL-6, TNF-α, and G-CSF after 8 weeks of cigarette smoke exposure; except for G-CSF, there was little difference between smoke-exposed *Ahr*^+/−^ and *Ahr*^−/−^ mice (*p < 0.05; ns = not significant). **(B)** Monocyte/macrophage- although CS increased BAL levels, there was no difference in CCL2 between *Ahr*^+/−^ and *Ahr*^−/−^ mice (**p < 0.01). **(C)** Lymphocytes- there was a significant increase in CCL20 in response to CS; there was significantly more CCL20 in the BAL of smoke-exposed *Ahr*^−/−^ mice (**p < 0.01; ****p < 0.0001). **(D)** Th17 response- IL-22 was unaffected by smoke exposure or AhR expression. Results are shown as means ± SEM (*n* = 4–5 mice per group); 2-way ANOVA followed by the Neuman-Keuls multiple comparisons test.

We also evaluated different types of cytokines in the BAL, including interleukins, tumor necrosis factor (TNF), chemokines and colony stimulating factors (CSF) after an 8-week cigarette smoke exposure. Although BAL cytokines were generally increased by cigarette smoke, there was little difference between *Ahr*^+/−^ and *Ahr*^−/−^ mice (Figure 2). These included cytokines associated with neutrophil recruitment such as CXCL1, CXCL2 and IL-6 (Figure 2A). G-CSF was significantly higher in smoke-exposed *Ahr*^+/−^ mice. Of the cytokines analyzed, only CCL20 was significantly higher in smoke-exposed *Ahr*^−/−^ mice (Figure 2C). There was no increase in IL-22 with cigarette smoke (Figure 2D).

**Figure 3:**
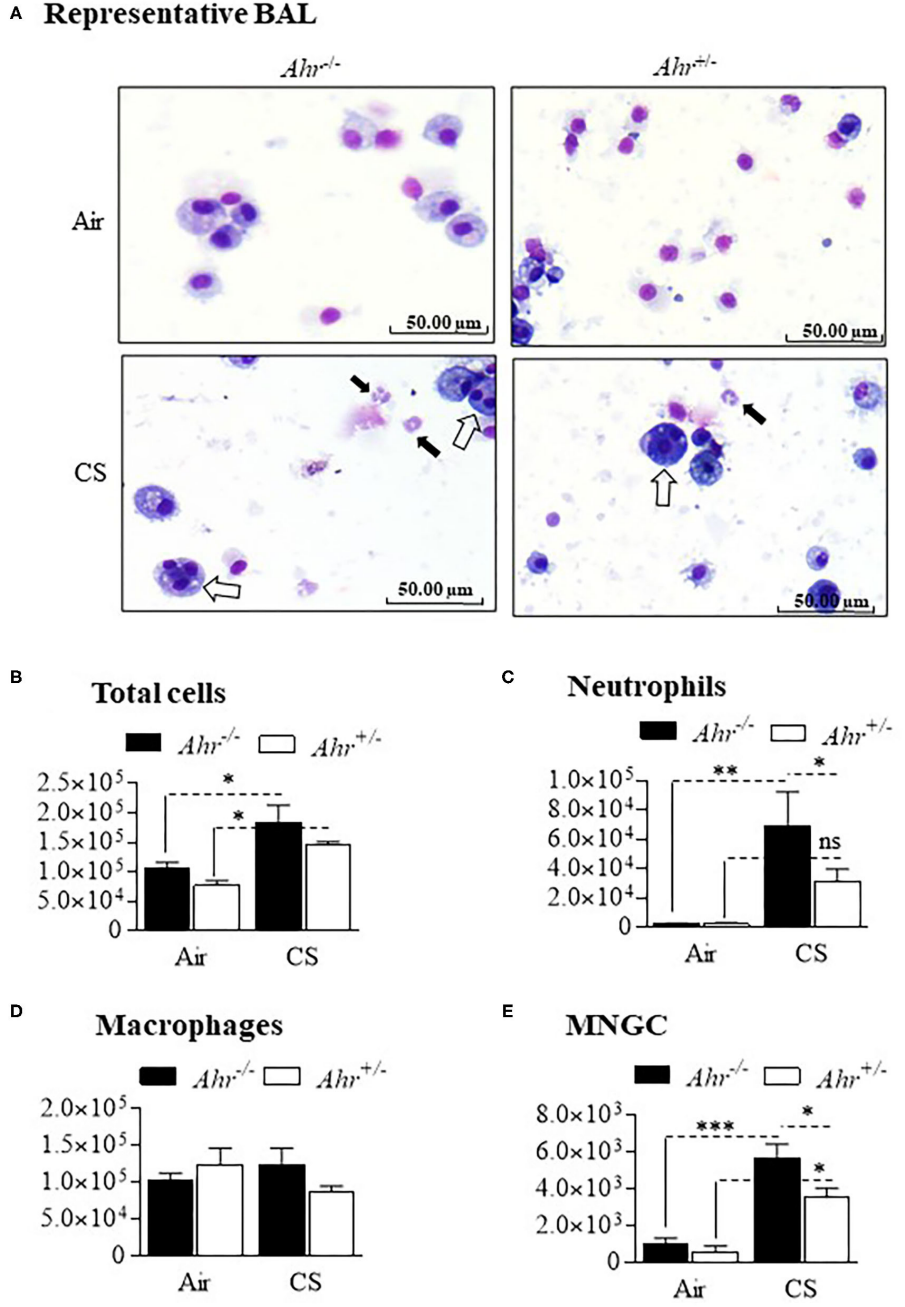
Exposure to CS for 4 months causes heightened pulmonary inflammation characterized by the continued presence of MNGC. **(A)** BAL Cells- Representative images of cells from the BAL of *Ahr*^+/−^ and *Ahr*^−/−^ mice exposed to CS for 4 months. Note the presence of neutrophils (*black arrows*) and MNGCs (*open arrows*). **(B)** Total Cells- There was a significant increase in cellularity in response to CS (*p < 0.05 compared to air-only exposure). **(C)** Neutrophils- CS induced an increase in BAL neutrophils in *Ahr*^+/−^ and *Ahr*^−/−^ mice (**p < 0.0001) that was higher in *Ahr*^−/−^ mice. **(D)** Macrophages- There was no change in the number of macrophages in response to CS. **(E)** MNGC- MNGCs were significantly increased in CS-exposed *Ahr*^−/−^ mice compared to both air-exposed *Ahr*^−/−^ mice (***p < 0.001) as well as CS-exposed *Ahr*^+/−^ mice (*p < 0.01). Results are expressed as mean ± SEM, *n* = 9–11 mice per group; 2-way ANOVA followed by the Neuman-Keuls multiple comparisons test.

The heightened inflammatory response persisted in the *Ahr*^−/−^ mice through 4 months of daily cigarette smoke exposure (Figure 3A). Although there was no significant change in the total number of cells (Figure 3B), there were higher number of neutrophils in *Ahr*^−/−^ mice exposed to cigarette smoke for 4 months (Figure 3C). While there was no difference in the number of macrophages between any of the treatment conditions (Figure 3D), there was a significant increase in the number of MNGCs in smoke-exposed *Ahr*^+/−^ and *Ahr*^−/−^ mice (Figure 3E). Moreover, there were also significantly more MNGCs in the smoke-exposed *Ahr*^−/−^ mice compared to *Ahr*^+/−^ mice exposed to a 4-month smoke regime (Figure 3E). These data show for the first time that the AhR prevents the formations of MNGCs, an enigmatic cell type linked to several pathologies.

**Figure 4:**
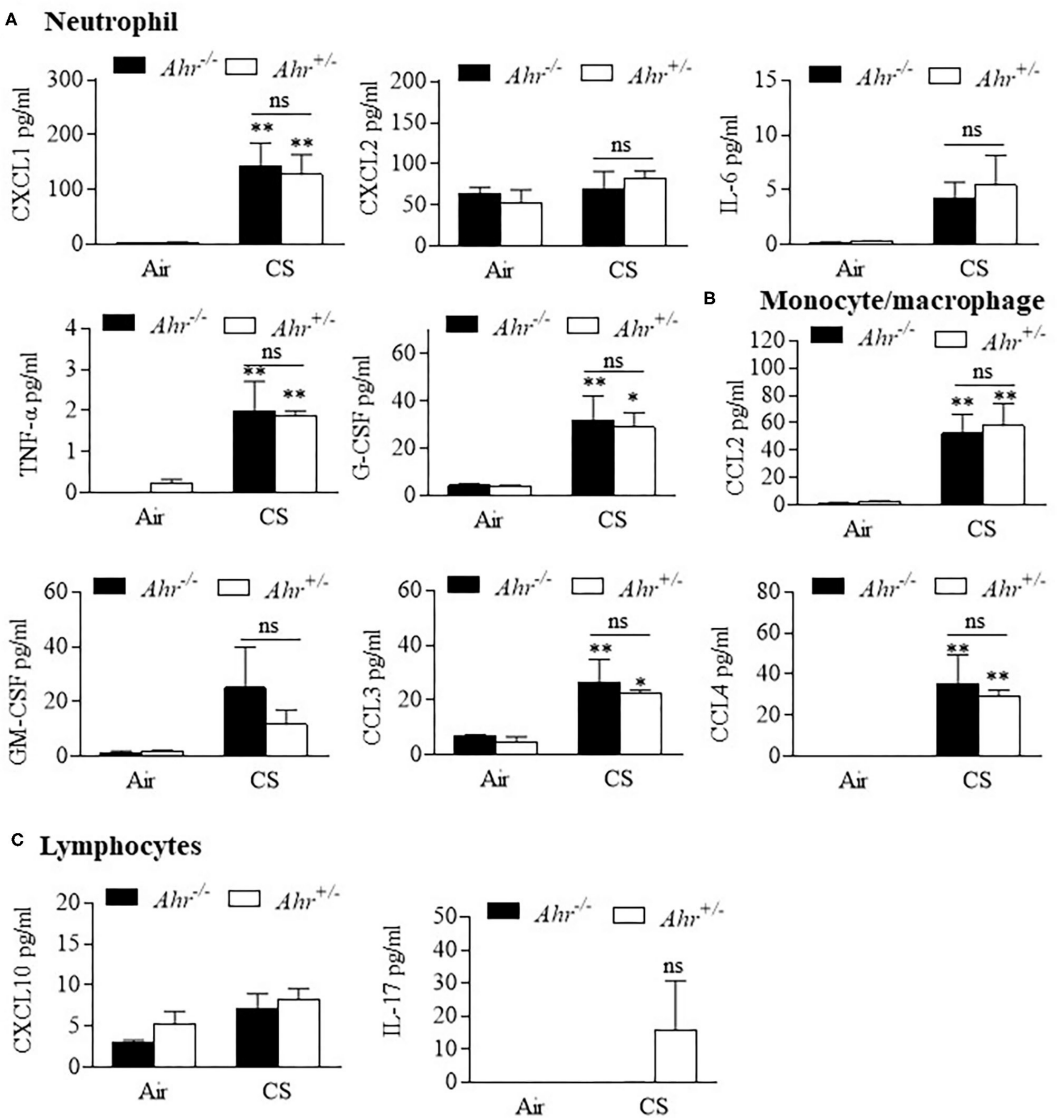
BAL cytokines are similar between chronic 4-month cigarette smoke-exposed *Ahr*^+/−^ and *Ahr*^−/−^ mice. *Ahr*^+/−^ and *Ahr*^−/−^ mice were exposed to a 4-month CS regime and cytokines analyzed in the BAL fluid by multiplex analysis. **(A)** Neutrophils- there was an overall increase in the levels of CXCL1, IL-6, TNF-α and G-CSF after 4 months of CS exposure (*p < 0.05, **p < 0.01 compared to corresponding air-control); there was no significant difference (ns) between smoke-exposed *Ahr*^+/−^ and *Ahr*^−/−^ mice. **(B)** Monocyte/macrophage- although CS generally increased BAL levels of CCL2, CCL3, and CCL4, there was no significant difference between *Ahr*^+/−^ and *Ahr*^−/−^ mice. **(C)** Lymphocytes- there was no significant change in CXCL10 or IL-17 in response to CS. Results are shown as means ± SEM (*n* = 4–5 mice per group); 2-way ANOVA followed by the Neuman-Keuls multiple comparisons test.

Finally, we evaluated BAL cytokines after the 4 month exposure regime and included additional cytokines to more comprehensively profile cytokine changes induced by smoke that may also be controlled by the AhR. Of the 31 cytokines analyzed, 8 were increased with chronic 4-month cigarette smoke exposure, including those associated with recruitment of neutrophils (Figure 4A) and monocyte/macrophages (Figure 4B) but not lymphocytes (Figure 4C). However, there was no difference in the levels of these cytokines based on AhR expression. Cytokines also evaluated but whose levels in the BAL were unaffected by cigarette smoke included eotaxin, IFN-γ, IL-1α, IL-1β, IL-2, IL-3, Il-4, IL-7, Il-9, IL-12p40, IL-12p70, IL-10, IL-13, IP-10, LIF, LIX, M-CSF, RANTES, and VEGF (data not shown). Thus, the AhR continues to emerge as an important protein involved in protecting the lungs against the deleterious effects of cigarette smoke, a prevalent respiratory toxicant that still causes significant morbidity and mortality around the world.

## Discussion

Although the AhR has been studied for decades, its physiological role has been elusive. Herein, we extend our previous finds to show the importance of AhR in the suppression of chronic cigarette smoke-induced pulmonary inflammation. We utilized two different exposure times- 8 weeks and 4 months- to mimic the scenario faced by people who smoke for decades. While cigarette smoke induces neutrophilia in the airways after ~5 days in C57BL/6 mice, increases in macrophage numbers become evident after 2 weeks and progressively increase through 6 months (D'hulst et al., 2005). We have previously reported that the *Ahr*-deficient mice exhibit significantly greater neutrophilia following acute smoke exposure (Thatcher et al., 2007), but whether the AhR can also protect against chronic exposure is not known. Herein, we report that chronic smoke-exposed *Ahr*-deficient mice exhibit an exaggerated inflammatory response typified by an initial increase in macrophages (8-weeks) and continued the presence of MNGCs (at 8-weeks and 4 months).

Macrophages undergo fusion with other macrophages to form MNGCs, a hallmark of chronic inflammation observed in tissues that incur persistent insults from foreign particles (Petersen and Smith, 2013), which is why they are often referred to as foreign body giant cells (FBGCs) (Brooks et al., 2019). FBGCs often form as a result of implanted material and are associated with their degradation. In addition, MNGCs are found in tuberculous where they may promote the inflammatory process. The role of MNGCs in tuberculous infection remains controversial, in that these cells may limit spread of the infection or promote tissue destruction (Losslein et al., 2021). Of relevance to our findings, MNGCs are also important sources of reactive oxygen species (Quinn and Schepetkin, 2009) and matrix metalloproteinase-9 (MMP-9) (Zhu et al., 2007) that may damage the lungs. MNGCs have recently been identified in the BAL of COVID-19 patients (Canini et al., 2021). However, identify of the precursor cells leading to the formation of MNGCs is unclear. Although macrophages and monocyte progenitors can give rise to MNGCs (Losslein et al., 2021), respiratory epithelial cells may give rise to MNGCs under certain conditions, such as viral infection (Lin et al., 2021). Although the physiological significance of MNGCs in chronic inflammation is not well-understood, our findings provide evidence for a protective role for the AhR in mitigating their formation and possible disease development.

Overall, these results continue to support a homeostatic role for the AhR in the maintenance of lung health (Guerrina et al., 2018). It also provides sufficient evidence to speculate that the AhR may lessen the susceptibility to COPD pathogenesis by attenuating chronic smoke-induced pulmonary damage by controlling the formation of MNGCs. Cellular pathways leading to the formation of MNGCs remain poorly understood. Therefore, these results provide evidence for the first time that the AhR is a biological pathway that reduces their formation in the lungs. Although we speculate that the ability of the AhR to prevent formation of MNGCs contributes to suppression of the emphysema-like phenotype (Guerrina et al., 2021), a limitation of our study is that we did not directly address whether MNGCs contribute to smoke-induced lung damage. We also did not evaluate how the AhR controls their formation or identify the cellular precursor(s). Given that AhR is expressed in multiple cell types, including structural and hematopoietic cells, and that AhR suppresses the influx of numerous immune cells to the lungs from both short and prolonged smoke exposures (Thatcher et al., 2007; de Souza et al., 2014; Rico de Souza et al., 2021), it is likely the cumulative effect of the AhR that ultimately prevent lung damage from cigarette smoke. Nonetheless, these results set the stage for further mechanistic studies aimed at addressing the contribution of MNGCs to the pathogenesis of diseases like COPD. Although smoking cessation remains the most effective strategy for slowing progression and reducing mortality (Tashkin, 2015), quitting smoking does not eliminate the risk of disease development. Continued investigation into the mechanistic basis by which the AhR controls inflammation may uncover new therapeutic targets to combat prevalent lungs diseases in both current and former smokers around the world.

## Data Availability Statement

The raw data supporting the conclusions of this article will be made available by the authors, without undue reservation.

## Ethics Statement

The animal study was reviewed and approved by all animal procedures were approved by the McGill University Animal Care Committee (Protocol Number: 5933) and were carried out in accordance with the Canadian Council on Animal Care.

## Author Contributions

NG, HT, and CB: data curation and analysis and project administration. CB: funding acquisition and supervision. NG and HT: investigation and methodology. NG, HT, CB, and DE: intellectual contributions, manuscript writing, review, and editing. All authors contributed to the article and approved the submitted version.

## Conflict of Interest

The authors declare that the research was conducted in the absence of any commercial or financial relationships that could be construed as a potential conflict of interest.
